# Biochemical and Biological Profiles of Bangladeshi Russell's Viper Snake Venom and Neutralizing Efficacy by Indian VINS Polyvalent Antivenom

**DOI:** 10.1155/jt/5464388

**Published:** 2025-09-29

**Authors:** Rubait Hasan, Hasanuzzaman Manik, Md Ataur Rahman, Jamiatul Husna Shathi, Md Tariqul Islam, Mohammad Shahangir Biswas, Kazi Md. Faisal Hoque

**Affiliations:** ^1^Department of Biochemistry and Biotechnology, Khwaja Yunus Ali University, Chouhali, Sirajganj 6751, Bangladesh; ^2^Department of Genetic Engineering and Biotechnology, University of Rajshahi, Motihar, Rajshahi 6205, Bangladesh; ^3^Department of Biochemistry and Biotechnology, University of Science and Technology Chittagong, Khulshi, Chittagong 4202, Bangladesh; ^4^Department of Public Health, Daffodil International University, Savar, Dhaka 1216, Bangladesh

**Keywords:** Bangladeshi Russell's viper, hemorrhage, histopathology, PLA_2_, procoagulant, VINS polyvalent antivenom

## Abstract

The Russell's viper (*Daboia russelii*) has recently become a significant threat to human life in Bangladesh. Given its wide distribution across South Asia, the venom characteristics and lethality can vary by region with different toxicological properties. Hence, we investigated the characteristics of Bangladeshi Russell's viper venom (BRVV) through SDS–PAGE profiling, reverse-phase HPLC analysis, along with assessments of phospholipase A_2_ (PLA_2_), edema-inducing, hemolytic, hemorrhagic, and coagulant activities, histopathology, and blood biochemistry, following established protocols. We also studied the neutralization efficacy of polyvalent antivenom from VINS Bio Products Ltd., India (VPAV) against BRVV. RP-HPLC analysis of BRVV displayed 15 peaks, and SDS–PAGE showed high-intensity protein bands within the 15–70 kDa range. The median lethal dose (LD_50_) for mice was found to be 0.33 mg/kg intraperitoneally (i.p.), and venom exposure resulted in neurotoxic symptoms such as limb paralysis, respiratory difficulties, and sluggishness. BRVV exhibited strong PLA_2_, procoagulant, hemorrhagic, indirect hemolytic, and edema-inducing activities but poor direct hemolytic activity. Venom administration also significantly increased levels of alanine aminotransferase (ALT), alkaline phosphatase (ALP), aspartate aminotransferase (AST), cholesterol, total protein, uric acid, blood urea nitrogen (BUN), and creatinine in mouse serum, indicating organ damage. Histopathological examination revealed cell vacuolization, congestion, hemorrhage, inflammatory infiltrations, and necrosis in venom-exposed tissues, validating the abnormal serum biochemistry. The neutralization study revealed that VPAV had limited efficacy against BRVV, suggesting the presence of venom proteins not fully neutralized by the antivenom. Altogether, these findings suggest that the Russell's viper is a medically significant venomous snake in Bangladesh, and VPAV is only partially effective in reducing the venom's toxic effects. Therefore, region-specific venoms must be considered in antivenom development for more effective treatment in envenomation cases.

## 1. Introduction

Snakebite envenomation is a significant cause of death and disability worldwide. Snakebite envenoming is a major neglected ailment of the 21^st^ century, despite its prevalence and significance as a public health problem [1]. In 2010, the World Health Organization (WHO) categorized it as a neglected tropical illness [2]. In 2017, WHO revived snakebite as a “neglected tropical disease” to highlight the dangers it poses to human health [3]. Around the world, there are between 0.4 and 2.5 million cases of envenoming due to snakebites yearly, with an estimated 94,000 deaths [3, 4]. South Asia has the most snakebite envenoming (121,000) [4]. Epidemiological research conducted in Bangladesh in 2016 revealed an annual envenomation incidence of 10.98 per 100,000 with an annual death rate of 1.22 per 100,000 [5].

Bangladesh is highly endemic to snakebites, with significant regional variations in snake species distribution and envenomation patterns. In Bangladesh, there are 82 species of snakes, 28 of which are venomous [6]. Among these, the dominant medically significant snake species responsible for envenomation-related deaths include the common krait (*Bungarus caeruleus*), greater black krait (Bungarus niger), Wall's Krait (Bungarus walli), monocled cobra (Naja kaouthia), Russell's viper (*Daboia russelii*), and Green pit vipers (*Cryptelytrops* spp.). *Bungarus caeruleus* is a neurotoxic snake responsible for over 50% of snakebite-related deaths in Bangladesh [7]. Envenoming caused by common krait typically occurs at night and results in rapid neurotoxicity, with untreated mortality rates reaching 70%–80% [8]. *Bungarus walli* and *Bungarus niger* contribute significantly to the complexity of krait envenomations, with *B. walli* alone accounting for approximately 40% of all reported krait bites [9]. The monocled cobra (*N. kaouthia*) also plays a major role, responsible for nearly 30% of venomous bites and associated with a 40% fatality rate due to neurotoxic effects and local tissue necrosis [10]. Among viperid snakes, Russell's viper, though historically rare, has shown a resurgence in Bangladesh, particularly in northwestern regions, exhibiting a high mortality rate among envenomed victims due to coagulopathy and renal failure [11, 12]. Green pit vipers primarily cause local swelling and coagulopathy; however, severe cases involving intracranial hemorrhage and renal failure have also been reported [9]. Nationally, snakebites result in an estimated 6000 deaths annually, with neurotoxic envenomation from kraits and cobras being the leading cause of mortality [13], while Russell's viper envenomation, though less frequent, poses a high lethality risk due to its complex pathophysiology and limited antivenom efficacy [9].

Russell's viper has been known to exist in Bangladesh for over a century, and wildlife scientists knew of their scarcity up until 2013 [14]; however, the recent explosion of these snakes has posed a danger to human life in the region. This viper thrives rapidly in intensive agricultural zones with ample rodent prey, notably in paddy fields and villages [11]. Historically, due to water shortages, Bangladeshi agricultural land was only used for crop production twice a year; the rest was abandoned. As irrigation technology advanced, farmers began cultivating three crops annually. This year-round availability of crops led to an increase in rat populations, providing snakes with abundant food and suitable breeding habitats. Russell's viper has been reported in 17 of Bangladesh's 64 districts, with Chapai Nawabganj and Rajshahi being the most affected. Between 2013 and 2016, 20 fatalities from Russell's viper bites were recorded in these two districts [14]. Over the past decade, 272 people in Bangladesh have died from Russell's viper bites, with a fatality rate of 50% among those envenomed (Dr Abdullah Abu Sayeed, August 31, 2021). It is now considered one of the most clinically significant venomous snakes in the country.

Snake venoms are a complex mixture of proteins and tiny chemical molecules [15]; multiple toxins have been reported from Russell's viper venom (RVV), which induce a variety of pathophysiological manifestations, such as variably coagulopathy, pain, swelling, myotoxicity, acute renal failure, neurotoxicity, tissue necrosis, hemolysis, hemorrhages, and pituitary infarction [11, 16]. Components of venom and lethal potency of the same snake species from various geographical locations often demonstrate distinct toxicological and antigenic characteristics [17]. For instance, Russell's viper envenoming cases in Sri Lanka caused neurotoxicity and myotoxicity [18, 19], whereas Eastern Indian Russell's viper bites produced spontaneous hemorrhage [20], and Southern Indian instances were neurotoxic and myonecrotic [21]. Because Russell's vipers live in a wide variety of habitats, their venom may reflect the differences in clinical presentations, emphasizing the need for venom characterization from every particular location. However, there have been no previous investigations about the biological characteristics of the Bangladeshi RVV (BRVV) toxins, their effects on organs, or blood biochemical parameters.

The WHO advises the use of antivenin as the only effective therapy for snakebites [2]. Moreover, this organization has also advised that antivenom production should utilize high-quality pooled venom from the local geographical population where it is intended to be used, as venom characteristics differ geographically. However, Bangladesh has still not raised any snake antivenom against local species. A Bangladeshi pharmaceutical company, Incepta Vaccine Ltd., only packages and markets antivenom, which is originally produced by VINS Bio Products Ltd., India [14]. All other antivenoms available in Bangladesh are directly imported from India and are derived only from Indian snakes, not from Bangladeshi snakes [6]. It has been noted that, depending on the degree of variation in the venom's composition, antivenom developed for one location is either partly effective or completely useless against the toxicity of venom from another region. For instance, owing to significant venom variations, antivenom inefficacy was reported in many studies [22–24]. Hence, the effectiveness of Indian VINS polyvalent antivenom against different biological features of local snakes in Bangladesh is a matter of question. Therefore, to determine the efficacy of Indian antivenom against various biological features of BRVV becomes essential.

In the current study, we have investigated the biochemical and biological features of BRVV and assessed the effectiveness of VINS polyvalent antivenom in neutralizing some of the venom's adverse effects.

## 2. Methods and Materials

### 2.1. Venom and Antivenom Collection

Venom from the *Daboia russelli* snake was obtained from the Snake Rescue and Conservation Centre (SRCC), Rajshahi, Bangladesh. The venom was subjected to a vacuum freeze dryer at −60°C for lyophilization, and the dried venom was preserved at −20°C until further use. The study utilized a polyvalent antivenom produced by VINS Bioproducts Ltd., Hyderabad, India (584 mg/vial; product was used before expiry). According to the label claim, 1 mL of the reconstituted antiserum can neutralize no less than 0.6 mg (dry weight) of *D. russelii* venom. 1 mL of the reconstituted solution contains 58.4 mg of antiserum. Given that 58.4 mg of antiserum neutralizes 0.6 mg of venom (equivalent to 0.01 mg (10 μg) of venom neutralized per 1 mg (1000 μg) of antivenom), it follows that at a 100:1 ratio (antivenom to venom, w/w), the antivenom should completely neutralize venom activities.

### 2.2. Ethical and Institutional Approvals

Healthy and pathogen-free male Swiss albino mice (20–24 g body weight) obtained from the animal house of the Department of Genetic Engineering and Biotechnology, University of Rajshahi, were used for the in vivo assays. The animals were maintained in ventilated cages in a pathogen-free room with controlled temperature (22°C–26°C). All procedures regarding animal treatment were carried out in accordance with the European Union Directive 2010/63/EU on animal welfare and approved by the Institutional Animal, Medical Ethics, Biosafety, and Biosecurity Committee (IAMEBBC) for experimentations on animals, humans, microbes, and living natural sources (license no: 34/320-IAMEBBC/IBSC), Institute of Biological Sciences, University of Rajshahi, Bangladesh.

### 2.3. Sodium Dodecyl Sulfate Polyacrylamide Gel Electrophoresis (SDS–PAGE)

SDS–PAGE of the crude BRVV was carried out using the technique of Laemmli [25]. Different concentrations (50–200 μg) of crude venom dissolved in PBS were loaded on the gel well of a 15% polyacrylamide gel and electrophoresed at 120 V using Mini-PROTEAN Tetra Cell (Biorad, USA) in a reducing environment until the top of the bromophenol blue dye reached the bottom. The gel was then dyed with a silver stain [26] to visualize protein bands. PageRuler Plus prestained protein ladders were used as standard molecular markers.

### 2.4. Reverse-Phase HPLC (RP-HPLC) Analysis

Three milligrams of lyophilized crude venom was dissolved in 0.05% trifluoroacetic acid (TFA) and 5% acetonitrile (ACN) and centrifuged at 10, 000 × g for 10 min at 4°C. The supernatant was then fractionated by a Discovery BIO wide Pore C18 (15 cm × 2.1 mm, 3 μm) column using an Agilent LC-1100 HPLC system. Proteins were eluted at a rate of 0.5 mL/min for 56 min employing a linear gradient of 0.1% TFA in water (Buffer A) and 0.1% TFA in 80% ACN (Buffer B) and detected at 280-nm wavelength.

### 2.5. Median Lethal Dose (LD_50_) Determination

The LD_50_ was calculated using Meier and Theakston's approach with modifications [27] by injecting (i.p.) different dosages (0.2 mg–1 mg per kg body weight) of freshly dissolved crude venom diluted in a volume of 200 μL PBS into six groups of mice (*n* = 10 per dose, 22–24 g). Only a PBS injection was given to the control group. The animal behavioral changes and survival period of each animal were monitored for 24 h, and the LD_50_ was estimated by probit analysis.

### 2.6. PLA_2_ Activity Assay

#### 2.6.1. Turbidometric Method

The turbidometric method [28] with a small modification was used to measure the phospholipase A_2_ activity of crude RVV. Briefly, one fresh chicken egg yolk was separated and immediately homogenized in 250 mL of physiological saline containing 0.02% sodium azide to prevent microbial contamination. The egg yolk suspension was stored at 4°C in sterile, airtight glass containers and used within 72 h of preparation. Before each experiment, the yolk solution was brought to room temperature and gently mixed to ensure homogeneity. As a stock solution, 2 mg of lyophilized venom was dissolved in 2 mL (1 μg/μL, 1:1 (w/v)) of 20 mM Tris-HCl buffer (pH 7.4). Different concentrations of venom (1.0–320 μg) were each incubated for 10 min at 30°C in a reaction mixture (yolk suspension and 20 mM Tris-HCl buffer) with an overall volume of 2 mL. The control used for this experiment was the mixture of egg yolk solution and Tris buffer. The Tris buffer was set as blank. The reduced optical density (OD) was recorded at 740-nm wavelength in a UV-vis spectrophotometer (Thermo Scientific, USA). The absorbance 0.01 decrease in 10 min on incubation means that 1 unit of PLA_2_ activity is increased. The experiment was replicated three times for each concentration.

#### 2.6.2. Agar Plating Method

PLA_2_ activity assay was also evaluated using the egg yolk agar plating method with modifications [8]. In brief, different concentrations of BRVV dissolved in Tris buffer were loaded into 6-mm holes in a 1% agar plate containing egg yolk solution prepared in normal saline, followed by 18 h incubation at 37°C. The diameter of the hydrolytic zone was measured in mm using the scale. The Tris buffer served as a negative control. The minimum PLA_2_ dose (MPD) was considered as the amount of venom that can cause a 17-mm-diameter clear zone [29].

### 2.7. Edema-Inducing Activity

The edema of mouse paws caused by BRVV was measured in a procedure described by [30] with minor adjustments. Various venom doses (3 μg–15 μg) dissolved in 20 μL normal saline were administered to the plantar surface of the mouse's right rear foot pad. 20 μL of saline was injected into the left foot pad to act as a control. After 45 min, the mice were euthanized (barbitone, 30 mg/kg, i.p.), and then both of their feet were cut off at the ankle so that they could be weighed separately. To calculate the edema ratio, the increased weight of the swollen limb is compared to the weight of the healthy limb. An edema ratio of 120% is considered the minimal edema dosage (MED) [30].

### 2.8. Direct and Indirect Hemolytic Activity

Hemolysis of red blood cells (RBCs) caused by BRVV was measured following the method of Das et al. with slight modification [30]. Fresh cow blood was obtained from the neighborhood butcher shop in a container having 0.11 M trisodium citrate (1 citrate: 9 sodium). The collected blood samples were centrifuged at 3000 rpm for 15 min using a Heraeus Multifuge X1R centrifuge (Thermo Scientific, USA) to isolate RBCs and obtain platelet-poor plasma (PPP), which was subsequently utilized within 4 hours of collection. Bovine RBC pellets separated from plasma were washed three times and dissolved in a 0.9% (w/v) saline solution at a final concentration of 10% (v/v). Various concentrations of crude venom (1–100 μg) were added to a reaction mixture containing 150 μL of 10% RBC in saline (final volume 2 mL) and placed for incubation for 60 min at 37°C. The incubated samples were centrifuged for 10 min at 5000 rpm, and the OD of the supernatant was detected at 540 nm using a UV–vis spectrophotometer (Thermo Scientific, USA). The amount of hemolysis induced by dH_2_O was considered to be 100%. The hemolysis percentage was calculated according to the formula of Tan et al. [11].

For measurement of indirect hemolysis, 20 μL of egg yolk solution was provided as the substrate to the reaction mixture during incubation, and hemolysis was calculated as that for the direct hemolysis calculation. The amount of venom required to elicit 50% hemolysis is known as the minimum hemolytic dose (MHeD) [11].

### 2.9. Coagulation Activity

In vitro coagulation assay of crude venom was measured using the modified technique of Das et al. [30]. Human blood was collected in citrated tubes (0.11 M trisodium citrate) at a 1:9 ratio (citrate: blood) from Rajshahi Medical College Hospital according to their medical ethical procedure. By centrifuging the blood at 5000 rpm for 20 min at 4°C, PPP was prepared. Various venom concentrations in 20 μL of PBS were preincubated with 300 μL of PPP at 37°C for 3 min. Clotting was started by introducing 40 μL of 200 mM CaCl_2_. The clotting time of PPP was determined using a coagulation analyzer (STAGO, France). The clotting time of blood samples containing only PBS, instead of venom, was considered the typical clotting time. The results are the mean ± standard deviation of three investigations. The minimum coagulant dose (MCD) is the amount of venom that induces coagulation within 60 s [11].

### 2.10. Hemorrhagic Activity

The hemorrhagic activity of venom was assessed using the technique of Kondo et al., with some modifications [31]. Various doses of venom (5–20 μg) dissolved in 30 μL of saline were intradermally injected into mice, while only saline was administered to control animals. After three hours, mice were sacrificed under barbitone (30 mg/kg, i.p.) euthanasia, and the dorsal surface skin was peeled off to examine the inner surface area of hemorrhaging. The venom dose that results in a hemorrhagic area 10 mm in diameter from the injection site is used to compute the minimal hemorrhagic dose (MHD).

### 2.11. Serum Biochemical Analysis

For serum biochemistry analysis, a dose near LD_50_ of BRVV (0.30 mg/kg) dissolved in 200 μL of normal saline (0.9% NaCl) was injected intraperitoneally into mice (*n* = 3). The control mice received only 200 μL normal saline. After 3 hours of venom injection, animals were given anesthesia, and blood samples were collected from the thoracic aorta of the treated and control groups by cutting the abdominal portion using scissors. After collection, the blood samples were left at room temperature for 30 min to allow clot formation. They were then centrifuged at 3000 rpm for 15 min at 4°C, and the resulting serum was carefully separated and subjected to the measurements of various biochemical parameters including alanine aminotransferase (ALT), aspartate aminotransferase (AST), alkaline phosphatase (ALP), total protein, blood urea nitrogen (BUN), uric acid, creatinine, cholesterol, HDL, LDL, and triglyceride using an auto bioanalyzer (Dimension Xpand Plus Integrated Chemistry System, Forchheim, Germany) and commercially available kits (Human, Germany).

### 2.12. Histopathology

To evaluate the effects of BRVV on mice organs, histopathological investigations were conducted [32]. Briefly, after blood collection, the mice's liver, kidney, intestine, and heart were collected, rinsed with normal saline, and gently dried using a filter paper. The tissues were then fixed in Bouin's solution—a mixture of picric acid, formalin, and acetic acid—for 18 h, followed by thorough washing in phosphate-buffered saline and storage in 70% ethanol. Subsequently, preserved tissues were dehydrated through a graded ethanol series, cut, embedded in paraffin wax, and sectioned into 5-μm-thick slices using a microtome. The thin sections were mounted on clean glass slides and stained using hematoxylin and eosin (H&E). Histological observations were performed under a light microscope (Optica, Italy) to assess structural changes in the tissue architecture.

### 2.13. Neutralization Studies

#### 2.13.1. Neutralization of Lethality

The effectiveness of VINS antivenoms was determined against the 2LD_50_ of BRVV [8]. In brief, various doses of antivenom were mixed with 2LD_50_ of venom (ratios ranging from 1:10 to 1:200, venom to antivenom, w/w) and incubated for 30 min at 37°C. After that, each combination of 200 μL was given intraperitoneally to mice; 10 animals were utilized for each dosage of antivenom. The same quantity of venom was given to control mice in the absence of antivenom. The mice had free access to water and food. Survival numbers were recorded after 24 h.

#### 2.13.2. Neutralization of PLA_2_ Activity

##### 2.13.2.1. Turbidometry

For the neutralization experiment of PLA_2_ activity of venom in the turbidometric method [33], different doses of VPAV were mixed with 1 μg BRVV (1:25–1:800, venom: antivenom, w/w) and preincubated for 30 min at 37°C to neutralize PLA_2_. One OD-adjusted egg yolk solution was added to this, and a UV–vis spectrophotometer was used to monitor the reaction's absorbance at 740 nm at 10-min intervals. The quantity of venom that causes a decrease in 0.01 absorbance units at 740 nm in 10 min is considered one unit of PLA_2_ activity. The activity of the crude venom without polyvalent antivenin was set to 100% in order to compute the percentage neutralization.

##### 2.13.2.2. Agar Plate

Neutralization of PLA_2_ activity was also measured by the egg yolk agarose gel plating method, following the modified method of Gutiérrez et al. [34]. The MPD of crude venom dissolved in Tris buffer, and different doses of VINS antivenom (1:25–1:800, w/w) were incubated at 37°C for 30 min. Then, the mixtures were loaded into the wells of agar plates for a further 18-h incubation at 37°C. Crude venom without antivenom served as a control.

#### 2.13.3. Neutralization of Procoagulant Activity

The neutralization efficacy of the procoagulant activity of BRVV by antivenom was assessed following the method of Das et al. [30] with minor adjustments. Briefly, the venom of MCD was preincubated with various ratios of antivenom (w/w) for 30 min at 37°C. To this, 300 μL of human PPP was added. The mixture was incubated for an additional 3 min at 37°C. Clotting was started by introducing 40 μL of 200 mM CaCl_2_. The clotting time of blood samples that only contain PBS, instead of venom, was considered the typical clotting time. Results are the mean ± SD of three experiments.

### 2.14. Statistical Analysis

Statistical analyses were performed using the Student's *t*-test following Prism (Version 8; GraphPad Software, San Diego, CA, USA). The results were expressed as the mean ± SD of three independent experiments, except for LD_50_ determination. The LD_50_ of the venom was expressed as means with 95% confidence interval (CI). The minimum doses of different venom activities were calculated by regression analysis. *p* value < 0.05 was considered statistically significant.

## 3. Results

### 3.1. SDS–PAGE Analysis of Crude BRVV

**Figure 1:**
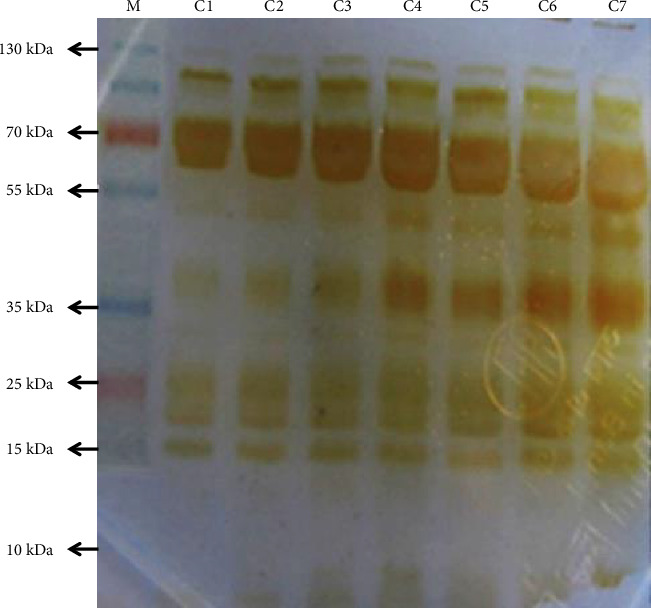
SDS–PAGE electrophoresis of the crude venom on a 15% polyacrylamide gel under reducing conditions. Lane M: PageRuler plus protein ladders. The molecular weight of standard proteins is marked in kDa. Lanes C1 to C7: BRVV venom samples at 50 μg (C1), 75 μg (C2), 100 μg (C3), 125 μg (C4), 150 μg (C5), 175 μg (C6), and 200 μg (C7), respectively.

The SDS–PAGE analysis of crude BRVV revealed multiple protein bands with a wide range of molecular weights, indicating the venom's complex composition (Figure 1). A high-intensity band was observed at approximately 70 kDa, with additional prominent bands at around 100, 35, 25, and 15 kDa. As the venom concentration increased (≥ 150 μg), additional faint bands became visible around 55 kDa and below 10 kDa, indicating the presence of lower abundance proteins at higher doses. The protein distribution suggests the presence of several protein families commonly associated with viperid venoms, such as phospholipases A_2_ (PLA_2_), serine proteinases, metalloproteinases, L-amino acid oxidase (LAAOs), and other enzymatic toxins. Moreover, faint bands detected near the 10-kDa region may correspond to low-molecular-weight proteins such as disintegrins, Kunitz-type serine proteinase inhibitors (KSPIs), snaclecs, and other small peptide toxins.

### 3.2. RP-HPLC Profile Analysis

**Figure 2:**
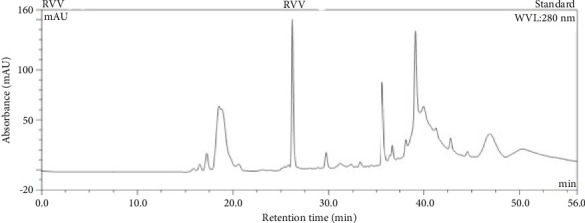
RP-HPLC analysis of BRVV. RP-HPLC was conducted on a discovery BIO wide pore C18 column pre-equilibrated with 0.1% (v/v) TFA. Approximately 3 mg of crude venom was added to the column and eluted with a linear gradient of 80% (v/v) ACN containing 0.1% (v/v) TFA over 56 min at a flow rate of 0.5 mL/min. The elution was monitored at 280 nm.

The RP-HPLC profile of BRVV is shown in Figure 2. In RP-HPLC analysis, the elution profile of crude BRVV displayed 15 peaks when venom was run for 56 min. A large peak was observed at 26 min, and another broad spectrum of proteins was observed at 35–48 min.

### 3.3. Biological Characterization of BRVV

**Figure 3:**
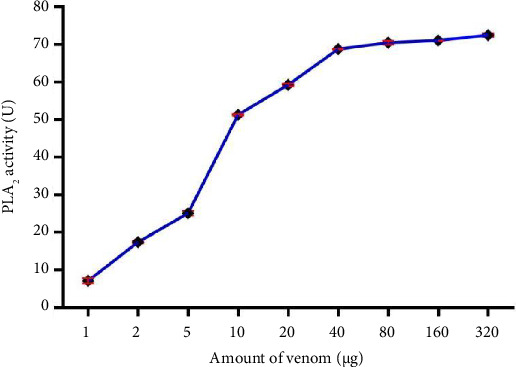
PLA_2_ activity of BRVV against egg yolk suspension in the turbidometric method. The decreased turbidity of the suspension was recorded at 740 nm after incubation at 30°C and compared with the control when the different concentrations of venom hydrolyzed the phospholipids of egg yolk lipoproteins. Data are presented as the mean ± SD of three replicates.

**Figure 4:**
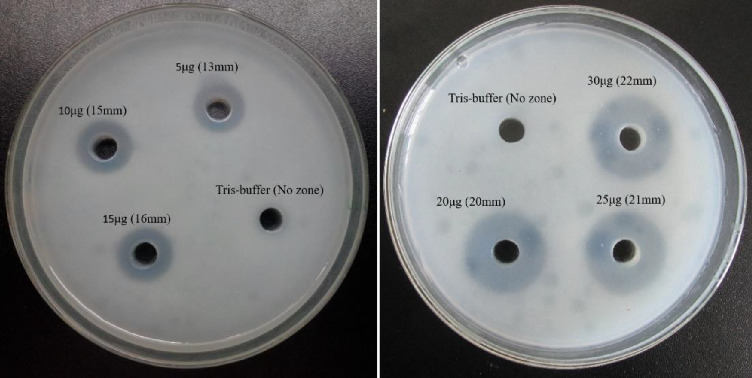
Photograph showing the PLA_2_ activity of BRVV against egg yolk solution in the agar plating method. Tris buffer represented no zone formation. A 5 μg venom dose induced a 13-mm transparent zone, while a 22-mm zone was developed by 30 μg venom.

**Figure 5:**
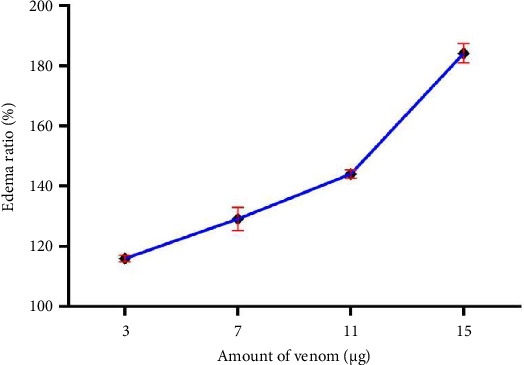
The edema-inducing activity of BRVV for different venom concentrations. The edema-inducing activity is expressed in the edema ratio. All values are expressed as the mean ± SD (*n* = 3).

**Figure 6:**
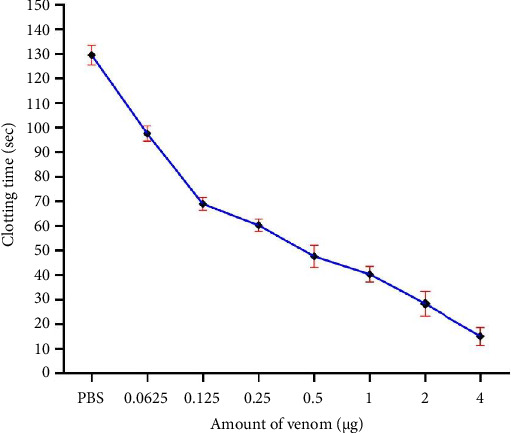
The procoagulant activity of BRVV on human plasma. The coagulation time of human citrated plasma was monitored in the presence of 200 mM of CaCl_2_, and PBS clotting time was considered as normal clotting time. Each point represents the average ± SD of three independent experiments.

**Figure 7:**
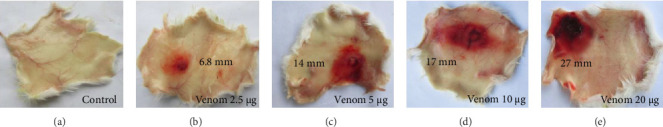
The hemorrhagic activity of Russell viper venom on the mouse skin. (a) The normal skin of control mice, and (b–e) hemorrhagic blood spots caused by various dosages of BRVV.

The biological activities of the crude BRVV are summarized in Table 1. The mice injected with venom exhibited severe neurotoxic signs/symptoms, such as hyperactivity immediately after injection, paralysis of the hind limbs, difficulty in movement, heavy breathing, sluggishness, and frequent drinking of water. The LD_50_ of BRVV was found to be 0.33 mg/kg when injected intraperitoneally into mice. PLA_2_ activity was measured by both turbidometric and agar plating methods. PLA_2_ activity of venom increased the transparency of cloudy egg yolk solution linearly with the increase in venom concentration (Figures 3 and 4). In the turbidometric method, 1 μg of venom induced 7.1 ± 0.7 U PLA_2_ activity, while MPD was measured at 17.49 ± 0.17 μg of venom in the agar plating method. The venom produced an increase in wet weight (edema) of the mouse foot pad after injection of venom linearly with the increased venom concentration (Figure 5). A venom of 4.75 μg is required to induce MED. In the case of the direct hemolytic activity study, no direct hemolysis was observed up to 5 μg of venom, but when the amount was increased up to 100 μg, it exhibited 12.74% RBC hemolysis. For indirect hemolytic activity, 40.93% hemolysis was observed for 1 μg of venom. The MHeD was measured to be 1.49 ± 0.21 μg venom. The BRVV exposed potent coagulant activity against human-citrated plasma (Figure 6). The clotting time at the concentration of 4 μg venom was 16.33 ± 4.73 s, while the normal clotting time was 129.67 ± 4.04 s. The MCD of crude venom was measured at 0.25 ± 0.13 μg. Intradermal injection of crude venom exposed strong hemorrhagic activity in experimental mice (Figure 7). Crude venom was found to have an MHD of 3.8 ± 0.28 μg.

### 3.4. Serum Biochemical Changes Caused by BRVV

Biochemical analysis revealed significant alterations in liver and kidney function markers in BRVV-treated mice compared to the control group (Table 2). Serum ALT and AST levels were markedly elevated (*p* < 0.01), indicating hepatocellular injury. A moderate but significant increase in ALP levels was observed (*p* < 0.05), accompanied by elevated total protein levels (*p* < 0.05), suggesting possible hepatic stress or a systemic inflammatory response. Kidney function markers, including BUN, uric acid, and creatinine, were significantly increased in the treated group (*p* < 0.05), indicating impaired kidney function. In the lipid profile, cholesterol levels were significantly higher in BRVV-treated mice (*p* < 0.05), while HDL and LDL levels remained statistically unchanged. A slight but nonsignificant reduction in triglyceride levels was observed. These biochemical findings collectively indicate that BRVV exposure causes both hepatic and renal toxicity in the treated mice.

### 3.5. Histopathological Effect of BRVV in Mice Organ

**Figure 8:**
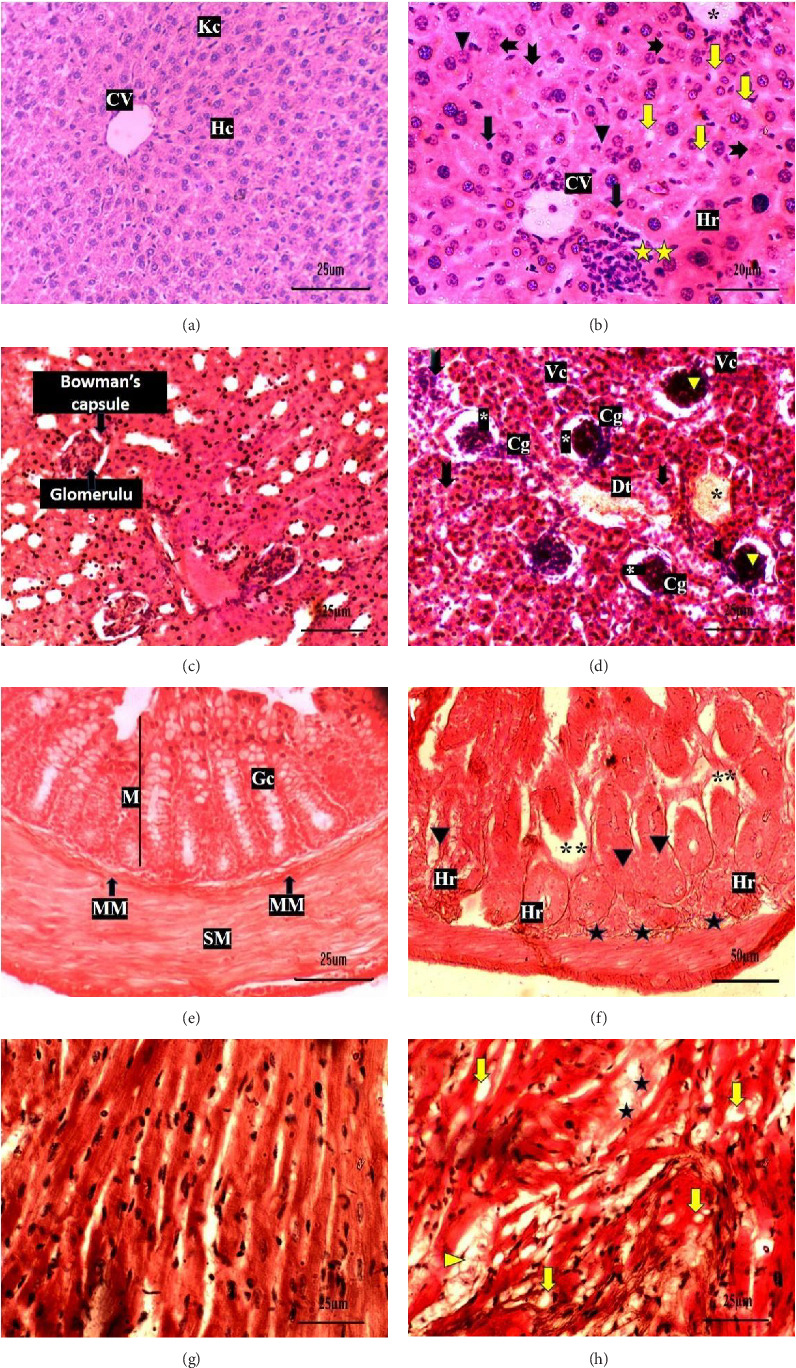
Histopathological sections of different organs in mice exposed to BRVV along with their respective controls, stained by eosin and hematoxylin. (a) In the control group, normal liver architecture was observed, including a clearly defined central vein (CV), organized hepatocyte cords (Hc), and visible Kupffer cells (Kc). (b) BRVV-treated groups exhibited pathological alterations such as central vein (^∗^) congestion, localized areas of inflammatory cell infiltration (^∗∗^), mild hemorrhage (Hr), cytoplasmic vacuolization (yellow arrow), karyorrhexis (arrowhead), karyolysis (arrow fork), and pyknotic nuclei (arrow). (c) Kidney sections from the control group displayed a normal renal cortex, characterized by intact glomeruli encircled by well-defined Bowman's space. (d) In the treated groups, the renal cortex exhibited pathological changes, including glomerular shrinkage and congestion (Cg), along with an abnormally widened Bowman's space (^∗^), necrosis in glomeruli (arrowhead), tubular necrosis (arrow fork), vacuolization in epithelial cells (Vc), congestion (black star), and dilatation of tubule (Dt). (e) Control group demonstrated typical morphology with healthy mucosa (M), submucosa (SM), and muscularis mucosa (MM). (f) The BRVV-treated group exhibited degeneration of the intestinal crypts, accompanied by epithelial shedding of the intestinal glands into the lumen (^∗∗^), degeneration of muscularis mucosa (black star), mild hemorrhage (Hr), and swelling and destruction of mucosal area (black arrowhead). (g) The untreated heart tissues show a normal appearance of cardiac muscle. (h) The venom-treated groups depict disorganization of myofibrils with the destruction of muscular striation in different areas, blood congested area (black star), cytoplasmic vacuolization (yellow arrow), and ruptured muscle fibers (yellow arrowhead).

Histological analysis was performed to assess structural alterations in the liver, kidney, heart, and intestinal tissues. In the control group, liver sections displayed normal hepatic architecture. The liver tissue showed well-organized hepatic lobules, each centered around a central vein and surrounded by radiating plates of hepatocytes. Kupffer cells were also clearly visible within the lobular framework (Figure 8(a)). In contrast, liver tissues from BRVV-exposed mice exhibited significant pathological changes, including central vein congestion, cytoplasmic vacuolization in hepatocytes, localized infiltration of inflammatory cells, hemorrhage, and some hepatocytes with signs of karyopyknosis, karyorrhexis, and karyolysis (Figure 8(b)). Histological examination of kidney tissue from control mice revealed normal renal parenchymal architecture, characterized by intact renal corpuscles with clearly defined glomeruli and well-organized proximal and distal convoluted tubules (Figure 8(c)). In contrast, kidney sections from BRVV-treated mice exhibited marked histopathological alterations. These included glomerular necrosis and shrinkage, accompanied by degenerated Bowman's capsule with a widened Bowman's space. Tubular structures showed epithelial degeneration, vacuolization in epithelial cells, congestion, and dilated distal tubules, which led to narrowing or complete loss of the tubular lumen (Figure 8(d)). Intestinal sections from control mice displayed normal histological architecture, with an intact mucosal layer featuring well-preserved intestinal crypts, abundant Goblet cells, well-defined submucosal glands, and intact muscular layers (Figure 8(e)). On the other hand, the venom-treated group showed significant histological alterations, including sloughing of the epithelial lining into the lumen, degeneration of the muscular layer and muscularis mucosa, and mild hemorrhage (Figure 8(f)). The cardiac tissue samples from envenomed mice depicted congestion of blood, cytoplasmic vacuolization, and disorganization of muscle fibers with the destruction of muscular striation (Figure 8(h)), while the cardiac tissue of control mice demonstrates normal configuration of the heart (Figure 8(g)).

### 3.6. Neutralization by Polyvalent Antivenom

**Figure 9:**
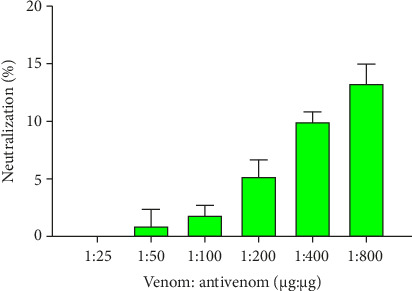
Neutralization of PLA_2_ activity of crude venom by VINS polyvalent antivenom in the turbidometric method. With the different amounts of antivenom, 1 μg of crude venoms was incubated at 37°C for 30 min, and then egg yolk solution was added. The increased turbidity of the suspension was recorded at 740 nm. Experiments were repeated thrice, and the mean values were used to plot the graph.

**Figure 10:**
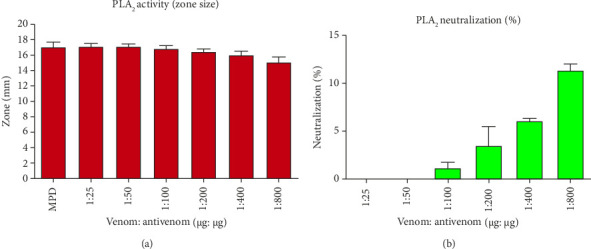
Inhibition of PLA_2_ activity of BRVV by VINS polyvalent antivenom. Neutralization of PLA_2_ activity of crude venom by VINS polyvalent antivenom was measured by the egg yolk agar plate method. Various antivenom concentrations (μg: μg) were applied to neutralize the minimum PLA_2_ dose (MPD). All data are presented as the mean ± SD (*n* = 3). (a) Antivenom activity was measured based on the decrease in a clear zone (mm) at various venom: antivenom ratios (μg: μg), where the zone is caused by PLA_2_ activity of venom. Decreased zone size indicates effective PLA_2_ neutralization. (b) Corresponding percentage neutralization of PLA_2_ activity by VINS antivenom.

**Figure 11:**
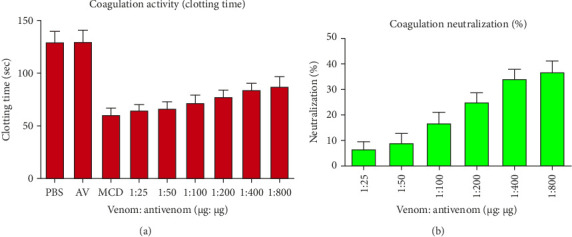
In vitro neutralization of BRVV-induced coagulation by VINS polyvalent antivenom. VINS antivenom dissolved in PBS was incubated with the minimum coagulant dose (MCD) of BRVV for 30 min at 37°C. The mixture was then incubated with human plasma for 3 min at 37°C, followed by the addition of 40 μL of 200 mM CaCl_2_ to initiate clotting. The data are shown as the mean ± SD (*n* = 3). Here, PBS = phosphate buffer saline and AV = antivenom. (a) Coagulation time (seconds) of BRVV-treated human plasma across different venom: antivenom ratios. Increased coagulation time indicates neutralization of the procoagulant effects of BRVV by VINS polyvalent antivenom. The control clotting time was determined using CaCl_2_-activated plasma with PBS alone. (b) Corresponding percentage neutralization of BRVV-induced coagulation by VINS antivenom at the same venom: antivenom ratios.

Different dosages of venom antivenom ratio (1:10, 1:50, 1:100, and 1:200, w/w) were used to evaluate the neutralization of lethality (Table 3). No mice treated with venom survived at dosages of 1:10 or 1:50. However, only 36.67% of treated mice survived with significant behavioral abnormalities at 1:200 doses. Therefore, the maximum dosage of VPAV employed in this study was ineffective in neutralizing the fatal action of BRVV. The neutralization of PLA_2_ properties of BRVV by VPAV is shown in Figure 9 (turbidometry) and Figure 10 (agar plate). At 1:25 (venom: antivenom, w/w), the polyvalent antivenom could not neutralize the PLA_2_ activity of the venom in both methods. Whereas, at 1:800 ratios, 13.34% (turbidometry) and 11.35% (agar plate) PLA_2_ activity neutralizations were noticed. BRVV-induced plasma clotted within 60.67 ± 2.52 s at MCD, whereas the normal clotting time was 130 ± 4.36 s. A dose-dependent increase in coagulation time was observed when venom was preincubated with the antivenom. The inhibition of the procoagulant effect of BRVV by VINS polyvalent antivenom is shown in Figure 11. At 1:25 (venom: antivenom, w/w), the polyvalent antivenom neutralized the procoagulant effect of BRVV only 6.74%, whereas, at 1:800 ratios, neutralization reached 36.85%.

## 4. Discussion

The pathophysiological impact of snakebite envenomation varies widely across species and even within species, owing to geographical variations and differences in venom compositions and biological activities [30]. Studies conducted on RVV from different regions have shown a remarkable regional diversity in the clinical symptoms of bites and venom compositions [17]. Therefore, it is crucial to comprehend the molecular and biological characteristics of snake venom from a certain region. Thus, this study characterized some biochemical and biological features of BRVV and the efficacy of Indian VPAV in attenuating venom-induced biological changes.

Snake venom consists of systemic toxins and hydrolytic enzymes, displaying distinctive protein-banding patterns upon electrophoresis. Additionally, the RP-HPLC profile reveals a unique chromatogram that is specific to certain species. Commonly documented toxins in RVV include PLA_2_s, serine proteinases and metalloproteinases (snake venom serine proteases [SVSPs] and snake venom metalloproteinases [SVMPs]), LAAOs, phosphodiesterase (PDE), snaclec proteins (SCLs), cysteine-rich secretory proteins (CRISPs), KPSI, and disintegrins [11, 16]. The SDS–PAGE analysis suggests the presence of multiple proteins in BRVV, including LAAOs, SVSPs, SVMPs, SCLs, PDE, CRISPs, PLA_2_s, disintegrins, and KPSI, with bands ranging from 10 to 130 kDa. The BRVV has revealed some high-intensity protein bands at 70-, 35-, 25-, and 15-kDa regions showing a similar banding pattern to that reported for Indian West Bengal RVV [33], while faint yet detectable lower molecular protein bands near 10 kDa at higher protein loads were noticed, as seen in the RVV venom of Karnataka (India) [33] and that of Sri Lankan origin [11]. Our RP-HPLC chromatogram displayed 15 distinct peaks across the gradient. These peaks likely represent diverse venom components: early-eluting fractions may contain low molecular weight, hydrophilic proteins such as PLA_2_, disintegrins, KPSI, and snaclec-type lectins, while later peaks likely include larger or more hydrophobic proteins like LAAOs, SVSPs, and SVMPs. The appearance of low molecular weight peaks in RP-HPLC aligns with the faint ∼10-kDa bands observed in SDS–PAGE, suggesting that such peptides are present but in low abundance. Although these smaller proteins were less sensitive in SDS–PAGE, their chromatographic visibility reinforces their presence in BRVV. Their faint presence on silver-stained gels may be due to their low abundance or inefficient staining of small peptides. However, these studies primarily identified various protein groups, their migration patterns on SDS–PAGE, and their elution profiles in RP-HPLC. So, further study is needed to confirm the identity of individual proteins in the gel using LC-MS/MS and to analyze the different fractions of the chromatogram by SDS–PAGE.

The determination of LD_50_ is essential for assessing venom toxicity. Venom lethality depends on the synergistic effects of various toxic components or a single potent toxin [35]. This study found that the intraperitoneal (i.p.) LD_50_ of BRVV is 0.33 mg/kg, similar to the LD_50_ (i.p.) of Kolkata RVV, which is 0.33 mg/kg [36]. A previous study determined intravenous (i.v.) LD_50_ values of BRVV as 3.69 μg/18–20 g mouse. However, reported LD_50_ values (i.p.) for RVV range from 0.33 to 6.9 mg/kg [17, 36, 37], reflecting geographical variations in venom composition. Based on the literature review, the venom of *Daboia russelii* from Bangladesh might be more lethal compared to other origins of RVV. However, a comparative lethality analysis under identical experimental settings is required to explore this in future studies.

The primary enzymatic family and main toxins in *D. russelii* venom are PLA_2_ enzymes [11], constituting 32%–63.8% of the total venom proteins [38, 39]. This enzyme group variably causes hemorrhage, hemolysis, neurotoxicity, coagulopathy, cardiotoxicity, myotoxicity, and edema [40]. In our study, the chicken egg yolk was used as the substrate to measure PLA_2_ activity because the clearance of egg yolk suspensions (clearance has happened due to hydrolysis of the phospholipids present in yolk lipoproteins) by venom is considered PLA_2_ activity [41], and BRVV cleared egg yolk solution showing concentration-dependent PLA_2_ activity. Localized swelling (edema) at the bite site is a common and early clinical sign of snakebite envenoming, mainly caused by PLA_2_ enzymes breaking down membrane phospholipids. Other enzymes like LAAO [42] and SVMPs [43] can also trigger edema. RVV has been reported to induce edema in mice paws [16, 17, 39], and this study observed a noticeable, dose-dependent edema following BRVV injection. The MED of BRVV was determined to be 3.30 μg, while RVV from a certain location in India showed a MED of 6 μg [44]. Prasad et al. demonstrated that the edema-inducing potential of RVV varies geographically [17]. Interestingly, BRVV at 100 μg showed 12.74% direct hemolysis of RBC, unlike RVV from Sri Lanka [11], which lacked direct hemolytic activity. Western and southern Indian venoms also displayed marked direct hemolytic activity [39, 45]. The low molecular weight proteins like PLA_2_ and KSPI, which are typically below 15 kDa, can directly damage RBC membranes through hydrolysis and cause direct hemolysis [16, 46]. PLA_2_s disrupt phospholipid bilayers, while KSPIs, although primarily serine protease inhibitors, may also contribute to membrane destabilization and hemolysis through interactions with ion channels or other cellular components. Their presence in the BRVV sample, as suggested by faint SDS–PAGE bands around 10 kDa, provides a mechanistic basis for the observed direct hemolytic activity, even in the absence of exogenous phospholipids. BRVV also induced strong indirect hemolytic activity (MHeD: 1.49 ± 0.21 μg) in the presence of exogenous phospholipids (egg yolk). Previous studies demonstrated indirect hemolytic activity of RVV [11, 39, 44, 47]. The indirect hemolytic action of the venom in the presence of egg yolk is due to the catalysis of phospholipids by PLA_2_ and the generation of lysophospholipids and free fatty acids, both of which are lytic in nature [30].

A significant clinical aspect of *D. russelii* envenoming is venom-induced coagulopathy, primarily mediated by the SVMP and SVSP families of proteins found in RVV [11]. RVV-X, a critical enzyme in SVMPs, activates Factor X (cleaving the Arg^52^-Ile^53^ bond) to Factor Xa, causing coagulopathy [11, 48], whereas RVV-V (arginine ester hydrolase), a serine proteinase, activates Factor V (cleaving the Arg^1545^-Ser^1546^ bond) to Factor Va, promoting coagulopathy [49]. BRVV exhibits strong procoagulant effects on human plasma in a dose-dependent manner, with an MCD of 0.25 ± 0.13 μg. This procoagulant activity is likely due to the presence of SVMP and SVSP enzymes in the venom. The procoagulant activity of RVV is well-documented, though it varies geographically [17, 33, 50]. However, anticoagulant properties were also observed in eastern Indian RVV [17]. Intradermal injection of viperid and crotalid venoms often causes hemorrhage, primarily due to SVSPs, SVMPs, and PLA_2_ enzymes [43]. Metalloproteases in viperid venom are key hemorrhagic agents, breaking down capillary endothelial cells and basement membranes [51]. This study found that BRVV induces hemorrhagic spots on mouse dermis with an MHD of 3.8 μg. The action is most likely mediated by hemorrhagic proteases. Aligned with our result, RVV from various Indian regions and Sri Lanka typically causes hemorrhage [11, 16, 17]. In contrast, some *Daboia* venoms lack hemorrhagic action [17, 51]. Venom-induced coagulopathy can worsen hemorrhage, leading to systemic bleeding and hypovolemic shock [52]. Additionally, LAAOs, disintegrins, and snaclecs, commonly found in RVV, can inhibit platelet aggregation, disrupting hemostasis and exacerbating hemorrhagic conditions [53–55].

The present study highlights that BRVV induces severe histopathological damage in multiple organs (liver, kidney, heart, and intestine) in mice, with significant alterations in serum biochemical markers. Intracellular enzymes release into the bloodstream following cell membrane damage, indicating organ-specific injuries. Histopathological alterations of envenomed mice livers suggest hepatic injury, consistent with previous reports of RVV-induced hepatic dysfunction [35]. The nuclear alterations (pyknosis and karyorrhexis) caused by BRVV may be due to increased cellular activity and nuclear interruption associated with the mechanism of venom detoxification [56]. Hepatocyte necrosis likely occurs due to phospholipase activity, which hydrolyzes cell membrane phospholipids [56]. Moreover, serine proteases and matrix metalloproteinases can break down the cellular matrix, destroying cells. These structural changes are consistent with the significant elevation of ALT, AST, ALP, and cholesterol levels in the serum. Elevated serum ALT, AST, and cholesterol levels indicate liver inflammation and necrosis, marking the liver as a primary target of venom toxicity. Renal histology revealed severe damage in the kidney tissues. Acute renal impairment after envenomation occurs as the kidneys work to eliminate toxins [57]. Asian RVV contains nephrotoxins like PLA_2_ and SVMP, causing glomerulonephritis, interstitial congestion, and tubular and cortical necrosis [58]. RVV-induced different renal abnormalities have been observed in mice [59] and envenomed patients [60]. These kidney damages correspond to increased BUN, uric acid, and creatinine, indicating impaired kidney function. RVV components are known to affect kidney biomarkers postenvenomation [61]. Elevated total protein content signifies intense protein degradation, increasing renal workload, and indicates kidney dysfunction. The elevation of these markers indicates impaired kidney function. The observed cardiac tissue damage could lead to the release of cardiac biomarkers, although specific markers for heart damage were not measured in this study. However, elevated AST levels partly reflect cardiac muscle damage in addition to liver injury [57]. Cardiac tissue analysis in envenomed mice showed cytoplasmic vacuolization, blood congestion, and disorganized muscle fibers, suggesting acute myocardial stress from venom cytotoxins. Intestinal damage can increase permeability and systemic inflammation, contributing to higher blood protein levels due to plasma protein leakage and their subsequent absorption into the bloodstream.

In Bangladesh, snakebite victims are mostly treated with Indian polyvalent antivenom. However, antivenoms developed for one geographic region are often ineffective in treating victims in another region [62, 63] and sometimes entirely fail to counteract the toxic effects of venoms [64, 65]. The study evaluated the neutralization efficacy of VPAV against BRVV, focusing on lethality, PLA_2_ activity, and procoagulant effects. The VPAV showed limited efficacy against BRVV. The highest antivenom doses tested could not neutralize 50% of venom activities; consequently, we could not measure the median effective dose (ED_50_: the antivenom dose (μg) at which 50% of venom activity is neutralized) and neutralization potency of antivenom against BRVV in the present study. Similarly, Pla et al. [23] found VINS and Premium Serums antivenoms ineffective against BRVV, with lower immunorecognition for BRVV toxins compared to South Indian, Sri Lankan, and Pakistani RVV. This poor recognition is likely due to low paraspecific recognition of specific PLA_2_ isotypes (Drk-a1, Drk-b1, and Drk-b2) and PIII-SVMP (Daborrhagin K) enzymes present as major toxins in BRVV but essentially absent in other regional venom proteomes [23]. Our findings also showed that VINS antivenom neutralized BRVV's procoagulant effects better than PLA_2_ activities, consistent with studies on Pakistani [38] and South Indian RVV [66]. Theoretical calculations suggest that neutralizing 3.9 mg of unique BRVV PLA_2_ (Drk-a1) would require at least 103 (Premium Serums) and 178 (VINS) vials of antivenom [23]. Nonetheless, it should be noted that while the current study employed a preincubation model to assess the neutralizing capacity of VPAV, this approach does not fully reflect clinical envenomation scenarios where antivenom is administered after venom injection. Therefore, future studies should incorporate postexposure antivenom treatment protocols to better mimic real-world clinical conditions and more accurately evaluate therapeutic efficacy.

The findings indicate that VINS antivenom is not fully effective against BRVV toxic activities, leading to deaths among Russell's viper-envenomed patients in Bangladesh despite antivenom treatment. A notable case involved an 18-year-old student from Nachole Upazila, Bangladesh, who was bitten by Russell's viper and died after receiving 50 vials of antivenom at Rajshahi Medical College with widespread manifestations of severe bleeding, rhabdomyolysis, irreversible shock, and renal failure [14]. In the last six years (2018–2023), 30% of Russell's viper-envenomed patients passed away after taking the Indian polyantivenom at Rajshahi Medical College. This inefficacy could be attributed to the overall lower immunoreactivity of antivenom toward BRVV, and the polyclonal antibodies developed against Indian Russell's viper differ significantly in a cross-reactivity pattern with BRVV. These results align with previous findings of varied venom immunoreactivity to the Indian polyvalent antivenom among Indian snakes from different locations [33, 67, 68]. Although snakebite-related deaths in Bangladesh are predominantly attributed to the direct effects of envenomation—such as coagulopathy, renal failure, neurotoxicity, or systemic shock—antivenom-associated complications cannot be overlooked. Clinical reports from Bangladesh indicate high rates of anaphylactic (64.51%) and pyrogenic (80.64%) reactions to Indian antivenom, which may contribute to fatal outcomes, particularly when large doses are administered [12]. These adverse reactions may be amplified by delayed administration of antivenom due to initial reliance on traditional healers or prolonged travel time to hospitals, use of ineffective antivenom, and a lack of premedication or supportive monitoring [10, 12, 69]. So, snakebite-related mortality may result not only from the primary pathophysiological effects of venom but also, in some cases, from antivenom-induced adverse reactions or inadequate neutralization of venom toxins. However, the actual causes of snakebite-related mortality in Bangladesh remain underexplored and warrant a comprehensive investigation.

Although concentration-dependent partial neutralization at high antivenom concentrations suggests some specific interactions between BRVV toxins and VPAV—possibly due to many shared epitopes and conserved sequences in venom polypeptides from both geographic specimens—the observed poor efficacy highlights the need for region-specific solutions. ELISA-based immuno-profiling of BRVV proteins should be assayed for a better understanding of the immunoreactivity and to improve antivenom design. Given the geographic spread and high mortality of Russell's viper envenomation in Bangladesh, particularly in northwestern districts like Rajshahi and Chapai Nawabganj, and the poor efficacy of Indian polyvalent antivenoms against BRVV, the development of a monovalent antivenom targeting Bangladeshi *Daboia russelii* venom appears essential. Such an antivenom, tailored to the local venom proteome, would likely offer greater specificity, improved neutralization of key toxins (e.g., PLA_2_, SVMPs, and SVSPs), and reduced adverse immunological reactions due to lower protein loads. Monovalent formulations may also reduce the number of vials required, improving both safety and treatment efficiency. Further research should aim to develop more effective region-specific monovalent antivenoms, which should be prioritized within national snakebite management policies to improve clinical outcomes for envenomed patients.

## 5. Conclusion

As snake venom composition varies due to geographic isolation, prey diversity, and local adaptation, it is crucial to decipher the venom characteristics and assess antivenom efficacy locally. SDS–PAGE analysis reveals that BRVV contains major toxin groups that disrupt hemostasis. BRVV is fatal, strongly procoagulant, hemorrhagic, hemolytic, and edematous, consistent with Russell's viper envenomation. Partial neutralization by VPAV required substantial antivenom, increasing treatment costs and hypersensitivity risks. Future research should focus on developing more effective, region-specific monovalent antivenoms to improve treatment efficiency and reduce costs. The findings in this study may not directly reflect clinical outcomes in humans but can guide the management of BRVV cases. Additionally, clinical observations and assessments of antivenom efficacy in snakebite patients are crucial for correlating laboratory findings with real-world outcomes.

## Figures and Tables

**Table 1:** Biological activities induced by BRVV.

Parameters	Activity
LD_50_ (mg/kg)^a^	0.33 (0.26–0.41)
PLA_2_ activity in agar plate (MPD; μg)^b^	17.49 ± 0.17
Edema (MED, μg)^c^	4.75 ± 0.3
Direct hemolytic activity (100 μg)	12.74 ± 0.38%
Indirect hemolytic activity (MHeD, μg)^d^	1.49 ± 0.21
Coagulation (MCD, μg)^e^	0.25 ± 0.13
Hemorrhagic activity (MHD, μg)^f^	3.8 ± 0.28

*Note:* Except for lethality, where a 95% confidence range is used to show variability, the other values are expressed as the mean ± SD.

^a^LD_50_: Median lethal dose: the amount of venom (μg) that kills 50% of mice injected by the intraperitoneal route; 95% confidence limits are included in parentheses.

^b^MPD: Minimum phospholipase dose: defined as the amount of venom that causes a 17-mm diameter clear halo.

^c^MED: Minimal edema dosage: an edema ratio of 120%, where the increased weight of the envenomed swollen leg is compared to the weight of the healthy leg of mouse.

^d^MHeD: Minimum hemolytic dose: the amount of venom required to elicit 50% hemolysis of RBC.

^e^MCD: Minimum coagulant dose: the amount of venom that induces coagulation of human plasma in 60 s.

^f^MHD: Minimum hemorrhagic dose: the amount of venom that develops a blood spot of 10 mm diameter.

**Table 2:** Serum biochemical analysis of BRVV-treated mice.

Parameter	Control	Treatment
ALT (U/L)	43.97 ± 1.46	87.1 ± 6.8^∗∗^
AST (U/L)	68.5 ± 1.95	137.13 ± 7.25^∗∗^
ALP (U/L)	97.37 ± 2.41	126.43 ± 4.49^∗^
TP (g/dL)	5.53 ± 0.21	6.37 ± 0.32^∗^
BUN (mg/dL)	12.6 ± 0.3	18.23 ± 1.25^∗^
Uric acid (mg/dL)	4.3 ± 0.44	5.29 ± 0.37^∗^
Creatine (mg/dL)	0.64 ± 0.03	0.87 ± 0.04^∗^
Cholesterol (mg/dL)	77 ± 3.61	94 ± 6.56^∗^
HDL (mg/dL)	28.33 ± 1.53	26.67 ± 1.52
LDL (mg/dL)	21.67 ± 2.08	24.67 ± 1.53
Triglyceride (mg/dL)	135.67 ± 2.89	125 ± 3

*Note:* ALT = alanine aminotransferase; AST = aspartate aminotransferase; ALP = alkaline phosphatase. Data are presented as mean ± SD of three replicates. Level of significance compared with control: ^∗^*p* < 0.05, ^∗∗^*p* < 0.01.

Abbreviations: BUN = blood urea nitrogen, HDL = high-density lipoprotein, LDL = low-density lipoprotein, TP = total protein.

**Table 3:** Neutralization of lethality of crude BRVV by Indian VINS polyvalent antivenom.

Activity	i.p. LD_50_ (mg/kg)	Challenge dose (mg/kg)	% inhibition by polyvalent antivenom				ED_50_ (μg) of antivenom
			1:10	1:50	1:100	1:200	
LD_50_	0.33 (0.26–0.41)	2LD_50_	0	0	20 ± 10	36.67 ± 5.77	ND

*Note:* i.p. LD50: intraperitoneal (i.p.) median lethal dose (LD_50_). ED_50_, median effective dose: amount of antivenom in μg at which 50% of mice survived.

Abbreviation: ND = not determined.

## Data Availability

Data will be made available on request from the corresponding author.
